# Effectiveness and Utility of Virtual Reality Simulation as an Educational Tool for Safe Performance of COVID-19 Diagnostics: Prospective, Randomized Pilot Trial

**DOI:** 10.2196/29586

**Published:** 2021-10-08

**Authors:** Tanja Birrenbach, Josua Zbinden, George Papagiannakis, Aristomenis K Exadaktylos, Martin Müller, Wolf E Hautz, Thomas Christian Sauter

**Affiliations:** 1 Department of Emergency Medicine, Inselspital University Hospital Bern Bern Switzerland; 2 Centre for Health Sciences Education Faculty of Medicine University of Oslo Oslo Norway; 3 ORamaVR SA Geneva Switzerland; 4 Institute of Computer Science Foundation for Research and Technology Hellas Heraklion Greece; 5 Department of Computer Science University of Crete Heraklion Greece

**Keywords:** virtual reality, VR, simulation, medical education, hand hygiene, COVID-19, PPE, nasopharyngeal swab, protection, effectiveness, utility, diagnostic, testing, pilot study

## Abstract

**Background:**

Although the proper use of hygiene and personal protective equipment (PPE) is paramount for preventing the spread of diseases such as COVID-19, health care personnel have been shown to use incorrect techniques for donning/doffing of PPE and hand hygiene, leading to a large number of infections among health professionals. Education and training are difficult owing to the social distancing restrictions in place, shortages of PPE and testing material, and lack of evidence on optimal training. Virtual reality (VR) simulation can offer a multisensory, 3-D, fully immersive, and safe training opportunity that addresses these obstacles.

**Objective:**

The aim of this study is to explore the short- and long-term effectiveness of a fully immersive VR simulation versus a traditional learning method regarding a COVID-19–related skill set and media-specific variables influencing training outcomes.

**Methods:**

This was a prospective, randomized controlled pilot study on medical students (N=29; intervention VR training, n=15, vs control video-based instruction, n=14) to compare the performance of hand disinfection, nasopharyngeal swab taking, and donning/doffing of PPE before and after training and 1 month later as well as variables of media use.

**Results:**

Both groups performed significantly better after training, with the effect sustained over one month. After training, the VR group performed significantly better in taking a nasopharyngeal swab, scoring a median of 14 out of 17 points (IQR 13-15) versus 12 out of 17 points (IQR 11-14) in the control group, *P*=.03. With good immersion and tolerability of the VR simulation, satisfaction was significantly higher in the VR group compared to the control group (median score of User Satisfaction Evaluation Questionnaire 27/30, IQR 23-28, vs 22/30, IQR 20-24, in the control group; *P*=.01).

**Conclusions:**

VR simulation was at least as effective as traditional learning methods in training medical students while providing benefits regarding user satisfaction. These results add to the growing body of evidence that VR is a useful tool for acquiring simple and complex clinical skills.

## Introduction

The COVID-19 pandemic is a global health emergency that places massive demands on health systems and health care workers [1]. Proper use of hygiene and personal protective equipment (PPE) is paramount to prevent spreading of disease and contamination of health care workers. One possible reason for the high infection rate of COVID-19 is ineffective use of PPE. In Italy, up to 20% of health care workers were initially infected with the disease [2].

PPE recommendations from international organizations are largely consistent (eg, those from the US Centers for Disease Control and Prevention [CDC] and World Health Organization [WHO]) [3-6]; however, the actual use of PPE is not. Health care personnel in all professions and at all levels of training have been shown to use incorrect techniques for donning and doffing of PPE and hand hygiene [7-11]. The main reason appears to be inadequate training in correct PPE technique and lack of assessment of proficiency [7,11,12].

Simulation proves to be a powerful tool to test the accurate use of hygiene skills that are relevant for the treatment of patients with COVID-19 (ie, PPE and hand hygiene) [13]; nevertheless, there is still ambiguity regarding which training method works best. A recent Cochrane review of evidence relating to PPE and protection of health care staff exposed to contaminated body ﬂuids highlights the lack of robust evidence in this area [11].

Furthermore, education and training of health care personnel is difficult with social distancing restrictions in place and shortages of both PPE [2] and testing materials.

Virtual reality (VR) uses computer systems to generate realistic pseudoenvironments that provide users with visual, tactile, and auditory sensations, with the possibility of realistic interaction with the virtual environment [14]. Milgram and Kishino [15] referred to mixed reality (MR) as the technologies which involve the merging of real and virtual worlds.

VR simulation with the use of head-mounted devices (HMDs) can offer a multisensory, 3-D, fully immersive, and safe training opportunity, avoiding the restrictions of social distancing and material shortages [16,17]. Through the concept of immersion, sense of presence, and interaction with the virtual environment in a real-time and realistic manner, VR simulation can create emotional experiences that facilitate experiential learning, exceeding other 2-D learning modalities [18].

The value of VR in medical education has already been demonstrated for various tasks [16,19-30]. VR is often used for training skills of varying complexity, ranging from simple nursing skills (Foley catheter placement, gaining venous access [31]) to laparoscopic/endoscopic/endovascular skills [23,32] or complex surgical procedures [26-29,33,34]. It is suggested that skills training as a first step to acquiring a competency can be better taught with VR than with traditional learning methods, because VR allows for a more active and immersive learning experience [21].

Recent studies suggest that VR improves postintervention knowledge and skills of health professionals better than traditional education or other types of digital education [21,31,35]. VR offers several advantages, as it provides possibilities for flexible learning and self-learning, providing standardization, reproducibility, and stimuli control; it enables automated generation of data about the details of simulations, including performance measurements that can be used for research or to provide automated individualized feedback [24]. The simple novelty of interactive technologies themselves, such as VR, can improve student motivation [36]. The initial cost and effort of creating the program can easily be compensated by broad distribution [16,37], as VR training is gradually finding its way into the medical curriculum [38].

Only a few virtual or mixed reality simulations exist for training hand hygiene [39-42]. In addition, high-quality studies evaluating the effectiveness and long-term retention of a VR simulation compared to conventional training methods are lacking.

Therefore, we hypothesized that a VR simulation could be an effective and useful tool with high student satisfaction to teach COVID-19 diagnostics, and we performed a randomized pilot study in medical students to explore (1) the effectiveness of a fully immersive VR simulation versus a traditional learning method regarding a COVID-19–related skill set (ie, proper hand hygiene, proficiency in PPE use, and correct acquisition of a nasopharyngeal specimen) tested in a simulated clinical scenario, and, as a secondary outcome, its long-term effectiveness 1 month after training; and (2) media-specific variables influencing training outcomes, such as usability, satisfaction, simulator sickness, and the experience of presence and immersion.

## Methods

### Study Design, Setting, and Ethical Approval

This is a prospective randomized controlled pilot study, taking place at the emergency department of the Inselspital, University Hospital Bern, Switzerland [43], from September to November 2020.

The study population consisted of a convenience sample of medical students at the University of Bern. All participants attended on a voluntary basis; no remuneration was provided. Informed consent was obtained. Data were collected, analyzed, and stored in anonymized form.

The local ethics committee deemed our study exempt from full ethical approval (Business Administration System for Ethics Committees Req-2020-00889).

### Inclusion/Exclusion

The inclusion criteria were as follows: medical students (years 3-6 out of a 6-year curriculum) at the University of Bern.

The exclusion criteria consisted of unwillingness to participate or to provide informed consent.

### Baseline Investigations

#### Baseline Survey

A brief survey about sociodemographic factors; prior training; and experience in hand hygiene and PPE use, taking of respiratory samples (nasopharyngeal swab), and prior experience with VR was performed after enrollment.

#### Assessments/Measurements

**Figure 1:**
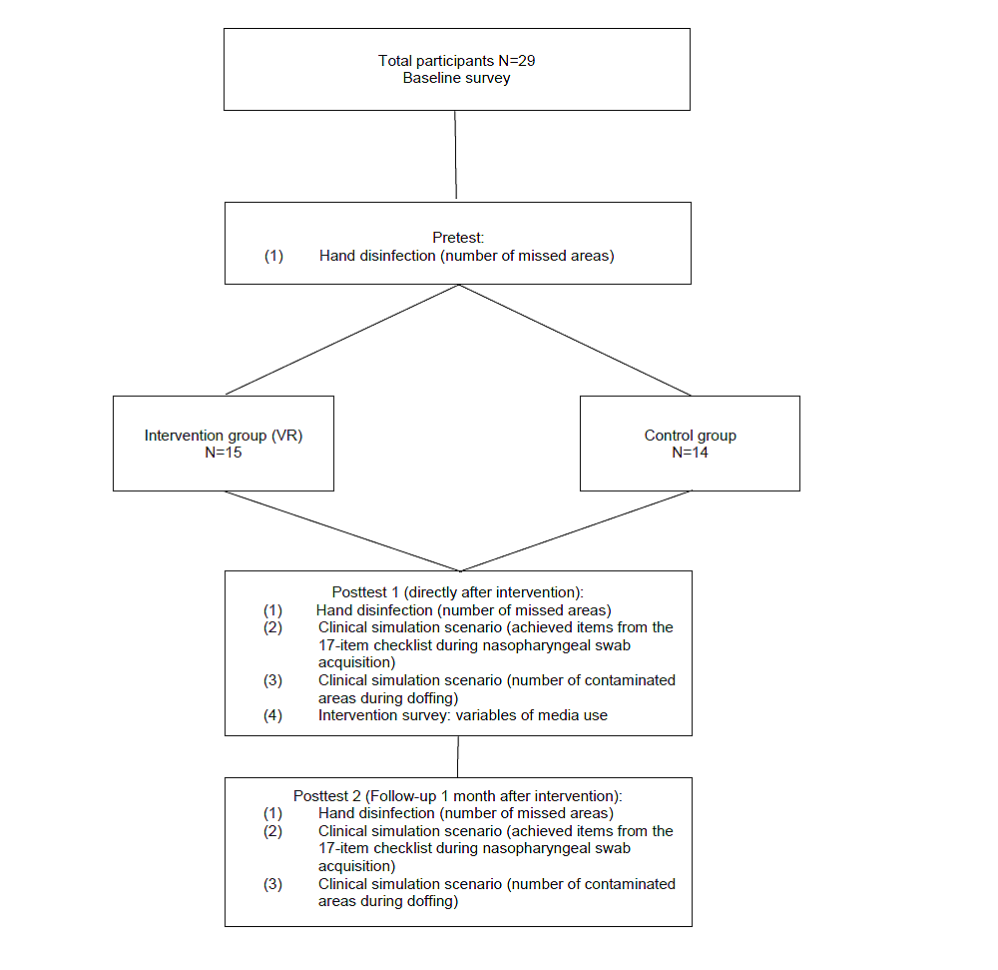
Flowchart of the study. VR: virtual reality.

We evaluated the performance of hand disinfection, taking a nasopharyngeal swab on a manikin, and contamination during doffing of PPE (Figure 1).

#### Hand Disinfection

**Figure 2:**
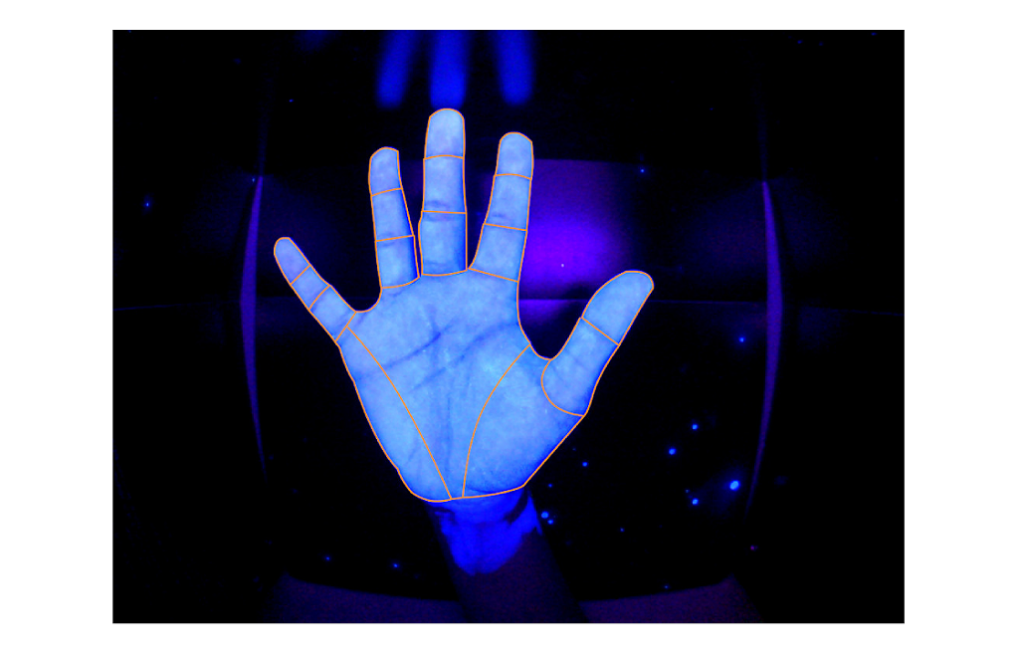
UV-light scanning of a perfectly disinfected palmar surface of the right hand.

**Figure 3:**
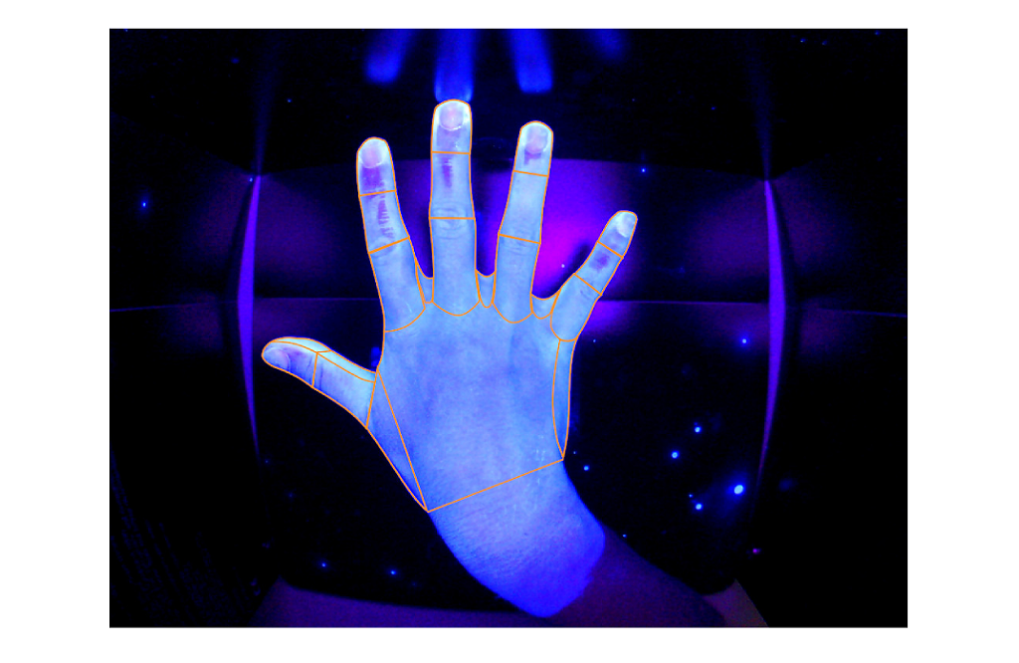
UV-light scanning of right hand dorsal surface. Missing areas of hand disinfection, right hand: digitus I, dorsal, distal phalanx; digitus II, dorsal, distal, and middle phalanx; digitus III, dorsal, distal, and middle phalanx; digitus IV, dorsal, distal phalanx; digitus V, dorsal, distal, and middle phalanx (total number of missed locations=8).

Hand disinfection performance was evaluated using a fluorescent marker (Visirub conc and Sterillium, Hartmann AG) and UV-light scanning was performed using the Derma Litecheck UV Multimedia device (KBD Ltd) at the time of enrollment (pretest), directly after the intervention (posttest 1), and 1 month after the intervention (posttest 2). Participants were blindfolded during the assessment and were unable to assess their results. A performance analysis scheme (documentation of missed locations; n=38 locations for each hand, with a total of 76 areas investigated) was developed by the institution’s infection control and medical educator team as adapted from Pan et al [44]. The outcome was the number of missed locations (range from 0 to 76; a lower number is better). Performance was supervised by an independent and trained rater, and the images were electronically recorded and analyzed according to the predefined scheme by a rater blinded to the intervention (Figures 2 and 3).

#### Obtaining the Nasopharyngeal Swab and Evaluating Contamination During Doffing

A simulation setup for conducting a nasopharyngeal swab for COVID-19 testing on a manikin (Little Anne, Laerdal Medical) using proper hand hygiene and PPE was installed.

The correct procedure of taking a nasopharyngeal swab sample as well as possible contamination while doffing were evaluated directly after the intervention (posttest 1) and 1 month after the intervention (posttest 2).

An independent and trained rater blinded to the intervention assessed each participant’s performance using a 17-item checklist adapted from [8,10] based on the CDC guidelines for PPE [3,6], WHO guidelines for hand hygiene [5], and international recommendations for taking a nasopharyngeal swab [6]; this checklist was developed by the institution’s infection control and medical educator team (Multimedia Appendix 1). The outcome was the number of points achieved on the checklist (range 0-17; a higher number of points indicated a better result).

Contamination during the procedure was evaluated using fluorescent lotion (Dermalux Testlotion S, KBD Ltd), which was applied to the participants’ hands, forearms, and torso before the doffing of PPE. After doffing, 10 areas (right hand, right forearm, right upper arm, left hand, left forearm, left upper arm, torso ventral, torso dorsal, neck, head/ears) were analyzed by UV lighting for contamination by an independent rater. The outcome was the number of contaminated areas (range from 0 to 10; a lower number indicated a better result).

### Intervention

Participants were randomized to either the intervention group (VR simulation) or control group in a 1:1 ratio using a computer-generated system.

### VR Simulation

**Figure 4:**
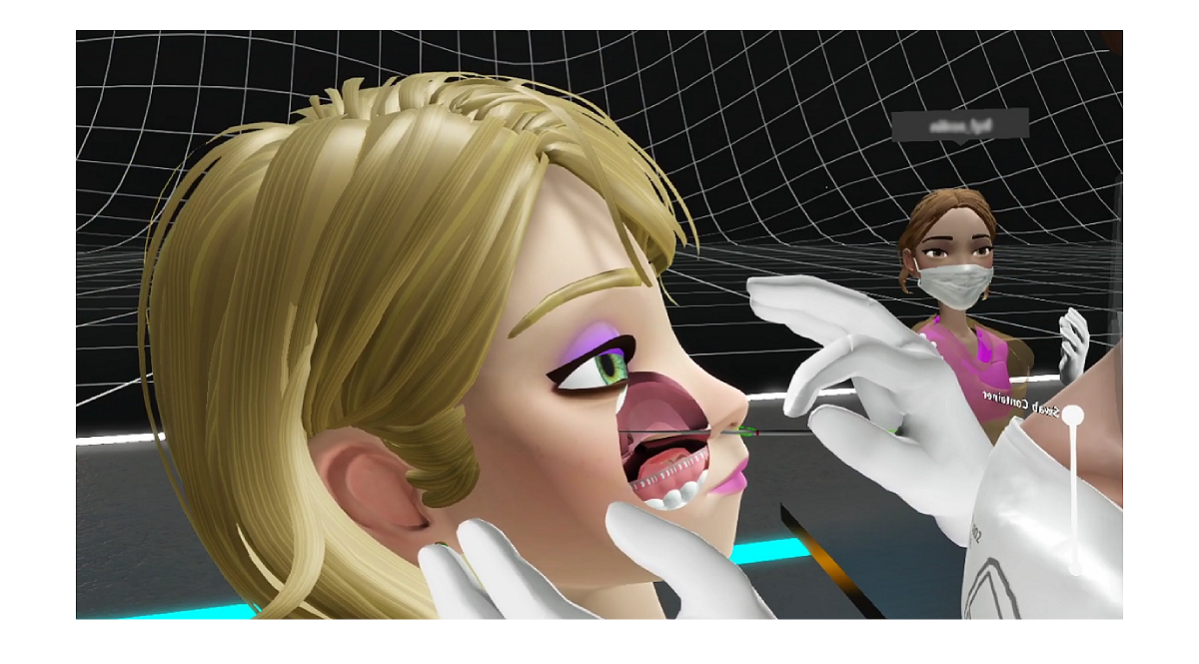
Screenshot of the virtual reality (VR) application, Covid-19 VR Strikes Back, showing the taking of a nasopharyngeal swab.

The intervention group was trained in COVID-19–related skills using the VR simulation (the Covid-19 VR Strikes Back (CVRSB) module, version 1.1.6), a software platform developed by ORamaVR SA, and the Oculus Rift S head mounted device and hand controllers (Facebook Inc). The proprietary ORamaVR software medical VR training application is available free of charge for all VR desktop and mobile HMDs [45] (Figure 4).

The participants performed two runs in the simulation using the single player modus.

### Control Group

The control group was trained using traditional learning methods: printed instructions and local instruction videos on COVID-19–related skills, ie, PPE donning and doffing, as well as formal videos on proper hand hygiene according to the WHO and on taking a correct nasopharyngeal sample [46].

### Intervention Survey

Both groups were evaluated regarding variables of media use according to established questionnaires.

Usability for both training modules was assessed using the After-Scenario Questionnaire (ASQ) [47], which assesses the ease of task completion, satisfaction with completion time, and satisfaction with supporting information on a 7-point Likert scale (total score ranges from 1, full satisfaction, to 7, poor satisfaction)*.*

The User Satisfaction Evaluation Questionnaire (USEQ) [48] contains 6 questions with a 5-point Likert scale to evaluate user satisfaction (total score ranges from 6, poor satisfaction, to 30, excellent satisfaction).

For the VR simulation, “visually induced motion sickness” was assessed with 4 items (nausea, headache, blurred vision, dizziness) according to the Simulator Sickness Questionnaire (SSQ) adapted from Kennedy et al [49] (total score ranges from 1, no simulator sickness, to 5, strong simulator sickness).

Presence and immersion in the virtual world was determined according to the 6-item questionnaire developed by Slater-Usoh-Steed [50] (total score ranges from 1, no immersion, to 7, full immersion).

### Statistical Analysis

Data were analyzed using SPSS, version 22 (IBM Corporation), and Stata 16.1 (StataCorp).

Baseline characteristics are presented as numbers and percentages or medians and interquartile ranges using descriptive statistics as appropriate.

The intervention and control group were compared regarding the baseline characteristics by chi-square test and Wilcoxon rank sum test as applicable.

The Wilcoxon rank sum test was used at a specific time point between the study groups for the comparison of all four outcome groups: (1) the number of missed areas during hand disinfection, (2) achieved items from the 17-item checklist during nasopharyngeal swab acquisition, (3) the number of contaminated areas during doffing, and (4) variables of media use. Within-group differences for different time points were tested using the Wilcoxon matched-pairs signed-rank test.

For all tests, a *P* value <.05 was considered significant. For this pilot study, no adjustment for multiple testing was performed. Furthermore, pairwise comparisons were favored over more complex analyses, such as mixed linear regression analysis.

## Results

### Sample Characteristics

In total, 29 students completed the study (control group, n=14; intervention group, n=15) (Figure 1). All students included completed the whole study. There were no dropouts. The baseline characteristics of the participants are detailed in Table 1. No significant differences were found regarding gender, mean age, educational level in medical school, need to wear glasses, previous experience with computer games, or previous experience with VR. Likewise, previous education and experience regarding hand disinfection, use of PPE, and taking nasopharyngeal swabs did not show any significant differences.

**Table 1:** Baseline characteristics of the study sample (N=29).

			VR^a^ group (n=15)	Control group (n=14)	*P* value	
**Sociodemographic factors**						
	Female gender, n (%)		9 (60)	9 (64)	.81	
	Age (years), median (IQR)		23 (22-25)	22.5 (22-24)	.56	
	**Year of medical school, n (%)**					.44
		3	1 (7)	2 (14)		
		4	14 (93)	11 (79)		
		5	0 (0)	1 (7)		
	Wears glasses, n (%)		8 (53)	8 (57)	.84	
	**Plays computer games regularly (Likert scale response^b^), n (%)**					.95
		1	10 (67)	10 (71)		
		2	4 (27)	3 (21)		
		3	0 (0)	0 (0)		
		4	0 (0)	0 (0)		
		5	1 (7)	1 (7)		
	**Uses VR regularly (Likert scale response^b^), n (%)**					
		1	15 (100)	14 (100)		
**Previous education and experience**						
	Previous education in hand disinfection, n (%)		14 (93)	11 (79)	.25	
	Previous education in PPE,^c^ n (%)		6 (40)	3 (21)	.28	
	Previous no. of swabs, median (IQR)		0 (0-0)	0 (0-0)	.54	
	**Uses PPE regularly (Likert scale response^b^), n (%)**					.36
		1	10 (67)	8 (57)		
		2	3 (20)	2 (14)		
		3	0 (0)	3 (21)		
		4	1 (7)	0 (0)		
		5	1 (7)	1 (7)		

^a^VR: virtual reality.

^b^Likert scale: 1, completely disagree, to 5, completely agree.

^c^PPE: personal protective equipment.

### Hand Disinfection

There was no significant difference in the number of missed areas during hand disinfection at baseline (intervention group: median 21, IQR 11-27, vs control group: median 20, IQR 14-21; *P*=.47) (Table 2). Both groups performed significantly better after training without a significant group difference (posttest 1) (median 7, IQR 4-14, in the intervention group vs median 10, IQR 6-14, in the control group; *P*=.34). For the secondary outcome, at posttest 2, again, no significant difference was noted between the intervention and control groups (median 14, IQR 8-17, in the intervention group vs median 11, IQR 7-16, in the control group; *P*=.74). In both groups, no significant difference was found between posttests 1 and 2 (intervention group, *P*=.11; control group, *P*=.25) (Table 3).

**Table 2:** Comparison between the VR group and the control group regarding hand disinfection, nasopharyngeal swab testing, and contamination during doffing.

		Values, median (IQR)			*P* value
		VR^a^ group (n=15)	Control group (n=14)		
**Number of missing areas during hand disinfection (out of 76 possible areas)**					
	Pretest	21 (11-27)	20 (14-21)	.47	
	Posttest 1	7 (4-14)	10 (6-14)	.34	
	Posttest 2	14 (8-17)	11 (7-16)	.74	
**Nasopharyngeal swab test** **score (out of 17 points)**					
	Posttest 1	14 (13-15)	12 (11-14)	.03	
	Posttest 2	14 (14-16)	14 (14-15)	.79	
**Number of contaminated body areas during doffing** (**out of 10 possible areas)**					
	Posttest 1	2 (2-4)	3 (1-4)	.64	
	Posttest 2	1 (0-2)	0 (0-1)	.18	

^a^VR: virtual reality.

**Table 3:** Comparison between posttests 1 and 2 for the VR group and the control group.

			Values, median (IQR)			*P* value
		Posttest 1		Posttest 2		
**Hand disinfection (number of missing areas)**						
	VR^a^ group	7 (4-14)		14 (8-17)	.11	
	Control group	10 (6-14)		11 (7-16)	.25	
**Swab test (score out of 17)**						
	VR group	14 (13-15)		14 (14-16)	.28	
	Control group	12 (11-14)		14 (14-15)	.02	
**Doffing (number of contaminated areas)**						
	VR group	2 (2-4)		1 (0-2)	.005	
	Control group	3 (1-4)		0 (0-1)	.003	

^a^VR: virtual reality.

### Nasopharyngeal Swab Acquisition

At posttest 1, the intervention group performed significantly better in taking a nasopharyngeal swab, scoring a median of 14 points on the 17-item checklist (IQR 13-15) versus 12 points (IQR 11-14) in the control group (*P*=.03) (Table 2). No significant differences between the groups were found after 1 month at posttest 2 (*P*=.79) for long-term retention as the secondary outcome.

The number of actual nasopharyngeal swabs performed in real life between posttests 1 and 2 did not differ between the groups (VR group, median no. of swabs 0, IQR 0-0; control group, median swabs 0, IQR 0-0; *P*=.56).

### Contamination During Doffing

No significant difference between the number of contaminated areas during doffing was found between the groups at both time points (Table 2). However, in both groups, a significant reduction of contamination was noted at posttest 2 compared to posttest 1 (intervention group: posttest 1, median contaminated areas 2, IQR 2-4; posttest 2, median contaminated areas 1, IQR 0-2, *P*=.005; control group: posttest 1, median contaminated areas 3, IQR 1-4; posttest 2, median contaminated areas 0, IQR 0-1, *P*=.003).

### Variables of Media Use

The results of the intervention survey regarding usability, satisfaction, simulator sickness, sense of presence, and immersion are detailed in Table 4.

**Table 4:** Variables related to media use.

	Values, median (IQR)			*P* value
	VR^a^ group (n=15)	Control group (n=14)		
After-Scenario Questionnaire score^b^	1 (1-3)	3 (2-4)	.002	
User Satisfaction Evaluation Questionnaire^c^	27 (23-28)	22 (20-24)	.01	
Simulator Sickness Questionnaire, abbreviated^d^	1 (1-3)	N/A^e^	N/A	
Presence and immersion according to Slater-Usoh-Steed^f^	5 (5-5)	N/A	N/A	

^a^VR: virtual reality.

^b^Range 1 to 7 (1=full satisfaction).

^c^Range 6 to 30 (30=optimal satisfaction).

^d^Range 1 to 5 (1=no simulator sickness).

^e^N/A: not applicable.

^f^Range 1 to 7 (7=full presence and immersion).

The ASQ revealed a significantly better result for the VR module (median score in the intervention group: 1, IQR 1-3; median score in the control group: 3, IQR 2-4; *P*=.002), as did the USEQ (median score in the intervention group: 27/30, IQR 23-28; median score in the control group: 22/30, IQR 0-24; *P*=.01).

The median score in the 4-item SSQ in the intervention group was 1 (IQR 1-3), thus revealing good tolerability of the VR simulation.

Presence and immersion in the virtual world according to the questionnaire of Slater, Usoh, and Steed was high (median 5 on a scale from 1 to 7, IQR 5-5).

## Discussion

In this study, we demonstrated that our VR simulation was at least as effective as traditional learning methods (video and written instruction) in training medical students in COVID-19–related skills—namely, the correct performance of hand hygiene, use of PPE, and taking of a nasopharyngeal swab specimen—and that it provides a benefit in user satisfaction.

### Effectiveness of Training

For the most investigated steps of the training, both educational methods improved performance to a similar extent without a significant difference. However, students in the VR group performed significantly better in acquiring a nasopharyngeal swab specimen on a manikin directly after the intervention than the control group, but this finding may be a result of multiple testing. Furthermore, the medians of the two groups only differed by 2 points on a 17-point outcome scale, and the IQRs overlapped.

There are few VR and MR simulations for training hand hygiene, and high quality evidence of their effectiveness is limited [39,40,42]. Shimada and colleagues [41] targeted preschool children, using a data glove instead of hand-held controllers to obtain the posture of user’s hand as a VR device. They used Leap Motion to obtain the posture of a hand, in contrast to our setting, which used only commercially available standard hardware. They found that their VR system was more effective than conventional hand hygiene instruction in a small group (n=12) of young children.

Performance of correct hand hygiene in our study was poor at baseline, in accordance with the existing evidence, stressing the need for effective instruction [7,11]. However, the performance significantly improved in both groups after the instruction, despite the limited technical possibilities to simulate the hand movements of disinfection with the need to hold the standard controllers in both hands while performing the correct movements. To minimize this limitation, participants could see the correct movements of their avatar’s hands (without the controllers) in a mirror.

This study adds to the body of evidence that VR simulation can help with the acquisition of simple skills, in combination with increased user satisfaction. Furthermore, our study highlights the necessity of strong collaborations between developers, users, and educators to ensure that these new technologies can complement and enhance existing educational curricula.

### Variables of Media Use

Satisfaction is considered to be one of the key components of usability [48]. The satisfaction of participants in the VR group measured by the USEQ was significantly higher than that of the participants in the control group. As most of our students were inexperienced in VR, the novelty effect may have added to the results. This effect consists of an increase in perceived usability of a technology due to its newness or the tendency for performance to initially improve when new technology is instituted, not because of any actual improvement in learning or achievement but in response to increased interest in the new technology. However, Huang and colleagues [51] state that novelty does not necessarily increase learning achievement. According to them, the increase of learning achievement is more dependent on a match between the learning content and the learning method. The embodied learning method in VR is particularly appropriate for instructing difficult knowledge and spatial knowledge [51]. It remains to be elucidated whether satisfaction with and efficiency of VR simulations will decrease over time as the technology becomes more widespread. However, with potentially increasing technological advances, a certain novelty effect will remain.

To make our VR training available to as many users as possible, we avoided using specialized hardware such as that used in many other studies of VR skill training. It could be speculated that this use of off-the-shelf controllers may reduce the realism of the VR simulation. However, we were able to demonstrate that a high degree of immersion and satisfaction could be achieved with our simulation even with standard hardware. This may be even more pronounced with future developments, such as hand tracking without the need for traditional controllers.

This “experience of presence” in VR, which could be demonstrated in our study, is known to correlate positively with training effectiveness [52,53].

### Skill Retention

In the follow-up after 1 month, there were no significant differences between the groups regarding any outcome. This cannot be explained by different exposures, as there was no difference in the mean number of swabs performed in real life in the meantime. One possible explanation is that the participants prepared more deliberately for the second appointment, as they might have suspected that it would involve a repetition of the first assessment (“assessment drives learning”) [54]. Most motor skills are lost over time, or at least the level of performance deteriorates, starting as soon as 1 day after training [55]. Maagaard et al [56] detected that the laparoscopic skills of novices acquired in VR simulator training deteriorated in a period between 6 and 18 months without further training.

We were able to show that the observed learning effect was maintained over the observed time frame of 1 month in both learning groups. Whether there will be a difference in skill decay between the two learning methods in the long run remains an important open question.

### Strengths and Limitations

Our study has several *strengths* to point out. First, the study assesses outcomes of direct clinical relevance, not surrogates such as performance on multiple-choice tests or user satisfaction only. Second, the study not only assesses the effect of training on performance gains but also includes a quantification of skill retention over time. Third, we compared the novel VR intervention to an established educational alternative rather than to no intervention.

In addition, attrition bias was nonexistent because all participants completed the study protocol without dropping out.

As a practical benefit, our VR simulation program is available for free; thus, program directors and educators are able to enhance their existing curricula with an effective novel adjunct or alternative, or replicate the study setting.

This study has several *limitations*, including its single center design, which restricts external validity. The number of participants in our study was limited due to the large logistical and human resources required to conduct the study during a pandemic. Therefore, the detection of small differences between training modalities is not possible with our study design. There is the possibility of selection bias, based on volunteer convenience sampling of medical students, as well as a possible performance bias, with allocation to the interventional group leading to higher motivation, satisfaction, and performance. The need to use hand-held controllers instead of hand-tracking might have further impacted the efficiency of the VR simulation; however, we wanted to apply technological equipment that is widely available.

Furthermore, the correlation of these findings to clinical, patient-oriented outcomes remains to be validated.

### Conclusion

To our knowledge, this is the first study using a VR simulation to train health care personnel in the correct use of hand hygiene, PPE, and taking a nasopharyngeal swab specimen and to compare the effectiveness to established traditional training (video and written instructions). VR simulation was at least as effective as traditional learning methods in training medical students while providing a benefit in user satisfaction. These results add to the growing body of evidence that VR is a useful tool for acquiring and maintaining simple and complex clinical skills.

## Appendix

Multimedia Appendix 1 Checklist used for performance assessment.

Multimedia Appendix 2 CONSORT-EHEALTH checklist (V. 1.6.1).

## Abbreviations

ASQ: After-Scenario Questionnaire
CDC: US Centers for Disease Control
CVRSB: Covid-19 VR Strikes Back
HMD: head-mounted device
MR: mixed reality
PPE: personal protective equipment
SSQ: Simulator-Sickness Questionnaire
USEQ: User Satisfaction Evaluation Questionnaire
VR: virtual reality
WHO: World Health Organization
