# Supplementary material for: Effectiveness and Utility of Virtual Reality Simulation as an Educational Tool for Safe Performance of COVID-19 Diagnostics: Prospective, Randomized Pilot Trial
Source: JMIR Serious Games. 2021 Oct 8;9(4):e29586. doi: 10.2196/29586 (PMC8510143; doi:10.2196/29586)
Supplement: Multimedia Appendix 1 [file games_v9i4e29586_app1.docx]

| **Item no** | **Item description** | **Comment** |
| --- | --- | --- |
|  | **DONNING (6 points)** |  |
| 1 | Initial hand disinfection |  |
| 2 | Don gown correctly | Must be fastened in waist and neck |
| 3 | Choose the right mask and don mask correctly | Fit flexible band to nose bridge, fit snug to face and below chin to cover nose and mouth |
| 4 | Don goggles |  |
| 5 | Don gloves correctly | Must extend to cover wrist of isolation gown |
| 6 | Correct donning sequence | Disinfection, gown, mask/goggles, gloves. |
|  | **SAMPLING (4 points)** |  |
| 7 | Label microbiological sample container | Before entering room |
| 8 | Swab introduction: correct position | Horizontal introduction, (correct distance (at least the distance between opening of nostril and ear lobe) |
| 9 | Swab rotation: correct duration | Rotate swab gently for 10 seconds. |
| 10 | No touching of the face/unprotected body area or adjustment of mask once contaminated without prior hand disinfection |  |
|  | **DOFFING (7 points)** |  |
| 11 | Doff gloves |  |
| 12 | Doff gown correctly | NOTE: also correct and all two points for item 11 and 12 if gloves and gown are removed in one step |
| 13 | Doff goggles correctly | Do not touch front of goggles |
| 14 | Hand disinfection after every step (gloves, gown, goggles) |  |
| 15 | Doff mask correctly | Grasp bottom ties or elastics, then the ones at the top, and remove without touching the front of the mask |
| 16 | Final hand disinfection |  |
| 17 | Proper doffing sequence | Gloves, gown (or both gloves+gown), goggles, mask, hand disinfection |
| Total score 17/17 |  |  |
